# Effects of repetitive transcranial magnetic stimulation on recovery in lower limb muscle strength and gait function following spinal cord injury: a randomized controlled trial

**DOI:** 10.1038/s41393-021-00703-8

**Published:** 2021-09-09

**Authors:** Søren Krogh, Per Aagaard, Anette Bach Jønsson, Krystian Figlewski, Helge Kasch

**Affiliations:** 1 Spinal Cord Injury Center of Western Denmark, Regional Hospital Viborg, Viborg, Denmark; 2 Department of Clinical Medicine, Aarhus University, Aarhus, Denmark; 3 Department of Sports Science and Clinical Biomechanics, University of Southern Denmark, Odense, Denmark

**Keywords:** Outcomes research, Spinal cord diseases

## Abstract

**Study design:**

Randomized sham-controlled clinical trial.

**Objectives:**

The objective of this study is to investigate the effects of repetitive transcranial magnetic stimulation (rTMS) compared to sham stimulation, on the development of lower limb muscle strength and gait function during rehabilitation of spinal cord injury (SCI).

**Setting:**

SCI rehabilitation hospital in Viborg, Denmark.

**Methods:**

Twenty individuals with SCI were randomized to receive rTMS (REAL, *n* = 11) or sham stimulation (SHAM, *n* = 9) and usual care for 4 weeks. rTMS (20 Hz, 1800 pulses per session) or sham stimulation was delivered over leg M1 Monday–Friday before lower limb resistance training or physical therapy. Lower limb maximal muscle strength (MVC) and gait function were assessed pre- and post intervention. Lower extremity motor score (LEMS) was assessed at admission and at discharge.

**Results:**

One individual dropped out due to seizure. More prominent increases in total leg (effect size (ES): 0.40), knee flexor (ES: 0.29), and knee extensor MVC (ES: 0.34) were observed in REAL compared to SHAM; however, repeated-measures ANOVA revealed no clear main effects for any outcome measure (treatment *p* > 0.15, treatment × time *p* > 0.76, time *p* > 0.23). LEMS improved significantly for REAL at discharge, but not for SHAM, and REAL demonstrated greater improvement in LEMS than SHAM (*p* < 0.02). Similar improvements in gait performance were observed between groups.

**Conclusions:**

High-frequency rTMS may increase long-term training-induced recovery of lower limb muscle strength following SCI. The effect on short-term recovery is unclear. Four weeks of rTMS, when delivered in conjunction with resistance training, has no effect on recovery of gait function, indicating a task-specific training effect.

## Introduction

Spinal cord injury (SCI) is associated with widespread disability for the individual due to detrimental effects on several body functions. In most individuals sustaining SCI, motor recovery is a priority goal of rehabilitation, as impaired motor function contributes considerably to loss of independence and reduced quality of life (QoL). For example, mobility limitations have been reported as major factors associated with reduced life satisfaction [1, 2], whereas impairments in mobility (walking-to-wheelchair transition) has been shown to reduce QoL and increase depression scores in SCI [3]. In addition, for individuals with motor-incomplete injuries, the ability to perform many activities of daily living, such as rising from chair or bed, walking, stair climbing etc., requires sufficient neuromuscular capacity and lower limb muscle strength to permit at least partial weightbearing. Therefore, rehabilitation designated to increase lower limb muscle strength is an important factor for improving mobility and functional independence following SCI. For example, Crozier et al. [4] have provided evidence that recovery of maximal muscle strength of the knee extensors is essential for functional ambulation following rehabilitation. Additional studies have supported this notion, while also identifying the importance of muscle strength in other muscle groups such as the hip flexors [5, 6]. Although a number of therapeutic interventions have been tested, resistance training (RT) remains the only method that, consistently, has proven capable of increasing maximal volitional muscle strength in SCI [7, 8]. Identifying novel therapeutic interventions that can augment or accelerate recovery of muscle strength in response to RT could therefore have a profound positive impact on the QoL in persons with SCI.

Repetitive transcranial magnetic stimulation (rTMS) is a non-invasive brain stimulation technique capable of increasing (facilitatory rTMS) or decreasing (inhibitory rTMS) cortical excitability, depending on the choice of stimulation parameters [9]. It is widely assumed that these effects are attributable to neuronal plasticity mechanisms such as long-term neuronal potentiation and depression (increased and decreased synaptic strength) [10], although a range of mechanisms have been suggested to contribute to rTMS-induced CNS plasticity [11]. A moderate body of literature has investigated the feasibility and efficacy of rTMS in SCI (for review on the use of non-invasive brain stimulation in SCI, see ref. [12]). Among the first reports, Belci et al. [13] published a case series describing the effects of five sessions of rTMS over a sham stimulation target (occipital cortex) compared to a therapeutical target (motor cortex). Following therapeutical rTMS only, four individuals with chronic tetraplegia showed significant improvements in the American Spinal Injury Association (ASIA) sensory and motor scores (now International Standards for Neurological Classification of Spinal Cord Injury (ISNCSCI)), electrophysiological measures, and timed pegboard test. In a larger trial that employed a comparable stimulation protocol and study population, Kuppuswamy et al. [14] subsequently reported similar findings for a host of functional and neurophysiological, but not clinical, outcome measures. In two randomized, sham-controlled trials, Kumru and colleagues [15] performed high-frequency rTMS combined with supervised gait training in groups of sub-acute SCI ambulators and marginal ambulators [16], and reported that rTMS had beneficial effects on the recovery in gait function and clinically assessed lower limb muscle strength. These findings suggest that rTMS—at least when combined with skill-based training—may increase motor skill reacquisition following SCI.

Whether rTMS can be used to facilitate recovery of maximal muscle strength following SCI is currently unknown. Recently, Leszczyńska et al. [17] have provided evidence that long-term facilitatory rTMS may improve motor unit function (increased amplitude of electromyographic signals) during maximal voluntary contractions (MVCs) and improve transmission of efferent neural impulses (increased motor-evoked potential (MEP) amplitude) to the upper extremities following SCI. In able-bodied individuals, Hortobágyi et al. [18] found that application of inhibitory (low-frequency) rTMS, but not sham rTMS, over hand primary motor cortex led to diminished RT-induced gains in MVC of the first dorsal interosseus following 4 weeks of training.

The main purpose of the present study was to investigate whether facilitatory rTMS applied over leg motor cortex as adjuvant therapy to lower limb RT (LL-RT) would lead to amplified gains in leg muscle strength, compared to sham stimulation, in a group of patients undergoing primary rehabilitation following SCI. As a secondary aim, we sought to investigate whether rTMS would be superior to sham stimulation for recovering gait function in a sub-group of individuals with various degrees of gait ability.

## Methods

### Participants

Participants were consecutively recruited from January 2019 to August 2020 at a specialized SCI neurorehabilitation center (The Spinal Cord Injury Center of Western Denmark, Department of Neurology, Regional Hospital Viborg, Denmark). Twenty-eight patients admitted for primary (initial) rehabilitation volunteered to participate in the study. After consent, participants’ medical records were checked for eligibility by specialist consultant neurologists. Inclusion criteria were as follows: age ≥ 18 years, first time SCI, motor-incomplete injury, and capable of participating in LL-RT classes. Exclusion criteria were as follows: history of multiple central nervous system lesions, recent head trauma, severe cerebral disorders, mental disorders, other neurological diseases, personal or familial history of epilepsy, and intracerebral implants of metallic or electronic origin. Participants were randomly block assigned (1 : 1) in blocks of two (based on age [young: 18–50 years or old: 51–75 years], sex [male/female], and The ASIA Impairment Scale (AIS)) [A, B, C, or D], using an online minimization randomization tool (www.sealedenvelope.com, Sealed Envelope Ltd, London, UK), to receive active (REAL) or sham (SHAM) rTMS. Participants were allocated immediately before the first rTMS session. All participants received detailed oral and written information about the study before giving informed, written consent.

### Study design

A blinded, randomized, sham-controlled trial design was applied. rTMS or sham treatment were performed daily (Monday–Friday) immediately before LL-RT classes (twice weekly) and lower limb physical therapy ([LL-PT] thrice weekly) for 4 weeks. LL-RT and LL-PT were part of the usual care activities and were supervised by physiotherapists who were masked to treatment allocation. LL-RT classes (60 min duration) followed the SCI Action International’s evidence-based scientific exercise guidelines for adults with SCI [19], which recommend strength exercises to be performed for three sets of ten repetitions for each major functioning muscle group, at moderate to vigorous (50–80% one-repetition-maximum) loading intensity. Training programs were individually tailored, but would include the following exercises: horizontal leg press, seated knee extensions, seated knee flexions, seated hip adductions, prone leg raises, and body weight-supported squats. LL-PT was based on the specific needs and capabilities of the participant, but would involve a combination of many of the following elements: stair climbing, balance and mobility exercises, overground- and body weight-supported treadmill training, functional electrical stimulation, and stretching/mobilization. Throughout the intervention period, subjects were engaged in additional clinical activities scheduled as part of their usual care, such as hydrotherapy, occupational therapy, activities of daily living training, and upper extremity RT classes. Assessments were carried out the day before the first rTMS session and the day after the last session, except for Lower Extremity Motor Score (LEMS) assessment, which was performed at admission and within 1 week of discharge. LEMS assessors (but no other assessors) were blind to treatment allocation.

### rTMS intervention

Participants were comfortably seated and reclined in a chair with a neck resting cushion. Stimulation was applied with a double-cone coil (The Magstim Company Ltd, Spring Gardens, Wales, UK) over bilateral leg motor cortex by positioning the center of the coil 0–2 cm anterior to the cranial vertex. The specific coil position for the individual session was determined by how noticeably it caused tingling sensations/twitch contractions in the thigh musculature (REAL participants only). The coil was powered by a Magstim Super Rapid^2^ Plus stimulator (The Magstim Company Ltd, Spring Gardens, Wales, UK). Stimulation parameters were as follows: 45 trains of 40 pulses @ 20 Hz @ 100% resting motor threshold (RMT) with 28 s between trains, totaling 1800 pulses over ~22 min. Sham rTMS was performed with a fixed coil position (2 cm anterior to the vertex), but with identical stimulation parameters and with the coil disconnected from the stimulator. Instead, a second, active coil (figure-of-8 coil, The Magstim Company Ltd, Spring Gardens, Wales, UK) was placed under the subject’s pillow, rotated away so that the magnetic field was directed into the headrest. This approach has previously been reported to successfully mask SCI persons undergoing sham rTMS [20]. RMT was determined as the minimum stimulator intensity capable of eliciting MEPs of ≥50 µV amplitude in ≥5 of 10 trials. MEPs were recorded by an electromyography system (Dantec Keypoint G3, Medtronic Dantec, Dantec Medical A/S, Skovlunde, Denmark) using Ag-AgCl surface electrodes placed in a belly-tendon montage over the abductor pollicis brevis on the dominant side. The decision to use a hand target for the RMT was made following pilot trials, where attempts at eliciting MEPs in various muscles of the lower limbs of individuals with SCI either proved unsuccessful or required such a high stimulation intensity that it would be ethically inappropriate for use in high-frequency rTMS.

### Outcome assessments

#### Maximal voluntary contraction

Subjects were seated 10° reclined in an isometric knee joint dynamometer (Knee Dynamometer, Science to Practice, Ltd, Ljubljana, Slovenia) and firmly fixated with Velcro straps across the midline of the stomach, over the knees and across the ankles. The rotational axis of the lever arm was aligned with the lateral femoral condyle and the force transducer/ankle pad at the distal end of the lever arm was positioned so that the distal edge of the pad was 2 cm above the medial malleolus. All trials were performed with the knee flexed at a 70° angle (0° = full extension) and signals were gravity-corrected (zero-shifted) before recording. The rotational axis of the force applied to the ankle pad was automatically captured by the equipment, based on the position of the pad, allowing for real-time torque calculation. After information about the test procedure was given, unilateral MVC for each side in each direction (knee extension, then flexion) was determined in the following way: subjects performed six submaximal muscle contractions (2 × 50%, 2 × 70%, 2 × 90% of maximal effort) as warmup and accustomization. After 1 min rest, three maximal attempts were performed separated by 45 s rest between trials. Each isometric contraction lasted between 3 and 6 s, and vigorous verbal encouragement was provided by the assessor (SK) during all trials. In situations where the highest peak torque was observed in the final trial, an additional attempt was performed. This procedure continued until the last attempt no longer yielded the highest value. Signals were captured using the supplier’s commercial software (ARS [Analysis & Reporting Software], Science to Practice, Ltd, Ljubljana, Slovenia). MVC was determined by the trial in which the participant produced the highest torque (Nm).

#### Lower extremity motor score

LEMS is the leg sub-score of the motor function grading system in the ISNCSCI worksheet [21] (2019 revised edition). The examination rates muscle strength subjectively on a six-point scale (0–5). For each patient, LEMS examinations were performed by the same experienced physician.

#### Gait function

Gait performance was assessed in a sub-group of ambulators (*n* = 8 each group) using the 10 m walking test (10MWT), Timed Up-and-Go test (TUG), and 6 min walking test (6MWT), all of which have been shown to be highly valid and reliable outcome measures to assess walking speed over a short distance [10MWT [22]], basic functional mobility and balance [TUG [23]], and walking endurance [6MWT [24]] in individuals with SCI. Walking tests were performed in a quiet, straight corridor on an even surface with 3, 10, and 30 meter tracks marked on the floor. For 10MWT, subjects were instructed to walk from the 0 m marker and cross the 10 m marker as “quickly and safely as possible.” For TUG, subjects were instructed to rise from the chair, walk to and round the 3 m marker without touching it, and walk back and sit down as “quickly and safely as possible.” The 6MWT was performed on a 30 m track and subjects were instructed to “cover as long a distance as possible in 6 min.” The 10MWT and TUG were performed three times with 1 min of seated rest between trials and the best performance was extracted for analysis. The 6MWT was performed once. Assistive devices were kept constant at PRE and POST testing.

### Statistical analysis

In order to detect an ES of 15% (*β* = 0.20, *α* = 0.05) for real rTMS (predetermined as the minimal clinically important difference (MCID)), based on previously published data on development of lower limb MVC following RT and neuromuscular electrical stimulation in SCI [25], *n* = 15 for each group was needed. Study data were collected and managed using REDCap electronic data capture tools [26] hosted at Aarhus University, Denmark. Statistical analyses were performed in STATA16 (StataCorp LLC, College Station, TX, USA). Two individuals (SK and KF) performed all data analyses separately with a data sheet that was coded for person identification and treatment allocation (simply coded as group 1 and 2), and then compared results afterwards. Assumption of normality was assessed using *Q*–*Q* plots (quantile–quantile plots) and variance homogeneity was assessed using *F*-tests. Repeated-measures analysis of variance (ANOVA) was performed for all outcomes, except LEMS and 10MWT. These failed the assumptions for ANOVA due to extreme outliers, which may bias the results. Each model included a between-subjects factor for treatment and a within-subjects factor for time (preintervention vs. post intervention). Analysis of covariance was performed (with baseline measures as covariate) before ANOVA, as groups appeared to differ at baseline on some measures. However, the original statistical model remained intact. For the remaining measures, within-group data were compared using paired two-sample *t*-tests for data with Gaussian distribution or Wilcoxon’s signed-rank tests for non-parametric data. Between-group comparisons were made using independent two-sample *t*-tests or Mann–Whitney *U*-tests. Parametric data are presented as means ± SD, whereas skewed data are presented as medians [interquartile range 25%;75%]. Two-tailed *p*-values are provided using a significance level of 0.05.

## Results

### Participants

**Fig. 1:**
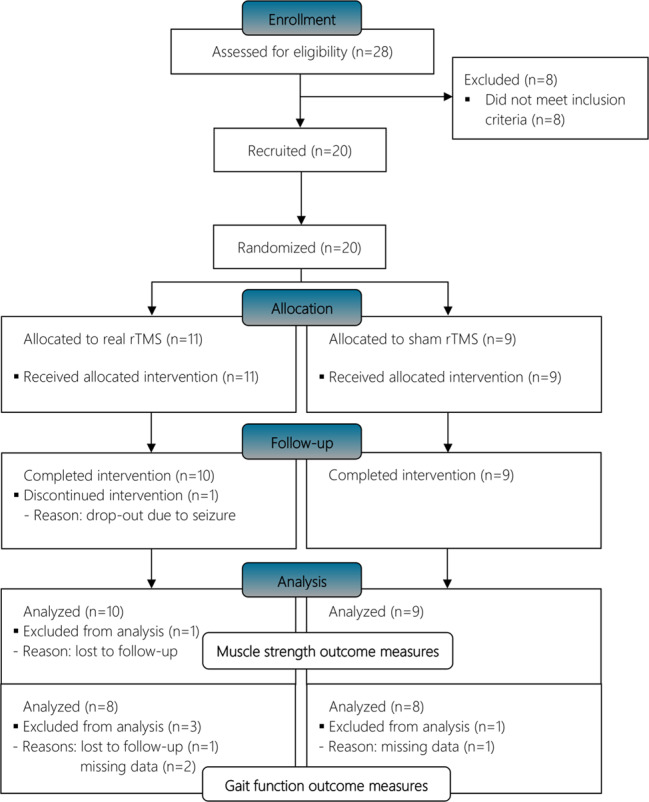
CONSORT flow diagram of enrollment, randomization, and follow-up. rTMS, repetitive transcranial magnetic stimulation.

Eight individuals failed to meet the criteria for participation, due to the presence of recent head trauma (*n* = 4), mental disorder (*n* = 2), cerebral disorder (*n* = 1), and motor complete injury (*n* = 1), leaving 20 participants to be randomized to receive active (REAL, *n* = 11) or sham (SHAM, *n* = 9) rTMS (cf. flow diagram in Fig. 1). One individual dropped out due to a seizure and was excluded from the data analysis. Out of 20 participants, 16 were ambulatory (*n* = 8 each group) and were analyzed for the effect of rTMS vs. sham stimulation on selected gait parameters (Fig. 1).

Baseline characteristics of the included study population are shown in Table 1. There were no significant differences between groups in terms of demographic variables, neurological level of injury, injury severity, time since injury, and global self-reported pain. In addition, there were no significant baseline differences between groups in any of the outcome measures. Exclusion of the dropout did not affect homogeneity between groups.

**Table 1:** Baseline characteristics of the study population.

Summary data				Individual data																		
	rTMS (*n* = 10)	Sham (*n* = 9)	*p*	P1	P2	P3	P4	P5	P6	P7	P8	P9	P10	P11	P12	P13	P14	P15	P16	P17	P18	P19
Age	57.1 ± 8.3	51.8 ± 12.1	0.28	35	36	47	70	60	59	43	55	61	48	68	56	60	51	64	58	57	42	67
Gender (M/F)	8/2	7/2		M	M	M	M	F	M	F	M	M	M	F	M	M	F	M	M	M	M	M
BMI	29.8 ± 6.7	27.4 ± 4.5	0.36	25.1	27.5	22.9	34.3	31.8	22.2	32.9	25.4	24.4	21.7	24.1	39.7	24.8	31.2	34.0	22.0	35.6	37.9	27.5
NLI^a^	10.5 ± 8.1	10.3 ± 7.8	0.96	L2	C4	C4	C8	C5	T12	C4	C2	L1	C5	C5	L2	C4	T10	T9	C2	C5	T3	T11
AIS classification^b^	3[2;3]	3[3;3]	0.55^d^	C	D	D	D	D	D	D	D	C	D	C	C	D	C	D	D	D	D	A
Time since injury (days)	91.3 ± 40.8	87.3 ± 69.5	0.88	81	31	58	71	253	26	46	97	123	133	115	90	30	105	41	158	72	110	59
Etiology (traumatic/non-traumatic)	3/7	3/6		T	NT	NT	NT	NT	T	NT	T	NT	T	NT	NT	NT	NT	NT	T	NT	NT	T
Self-reported global pain^c^	17.5 ± 17.5	24.0 ± 17.3	0.43	30	5	30	15	10	50	20	6	50	10	25	0	0	10	25	5	35	55	10

Reported as means ± SD or medians [95%CI]. P1–P9 received sham stimulation, P10–19 received real rTMS.

*AIS* ASIA Impairment Scale, *NLI* neurological level of injury.

^a^Calculated as C1 = 1, C2 = 2… S5 = 30.

^b^Calculated as *A* = 0, *B* = 1, *C* = 2, *D* = 3.

^c^Global self-reported average pain over the last 7 days (0–100-point numerical rating scale).

^d^Mann–Whitney *U*-test.

### Successfulness of masking

Immediately following conclusion of the post-intervention assessments, the participants were specifically asked: “What type of treatment do you think you have received in connection with this study?” and were given the following answer options: (a) “Real stimulation”; (b) “Sham stimulation,” or (c) “I don’t know”. Eight out of nine [89%] participants in SHAM were either uncertain or estimated that they had received real treatment. Six of ten [60%] REAL participants deemed themselves to have received real treatment, whereas four of ten [40%] were either uncertain or thought they had received sham treatment.

### Muscle strength

**Fig. 2:**
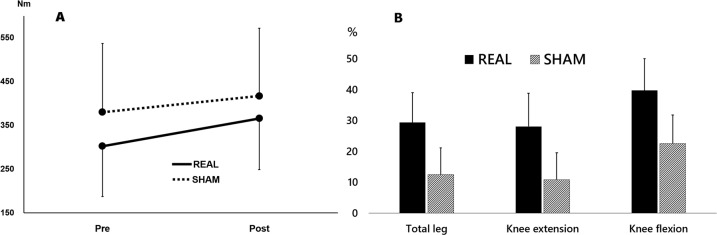
Developments in maximal leg muscle strength. **A** Total leg maximal voluntary contraction torque before and after four weeks of intervention.. Whiskers signify SD. **B** Relative gain in muscle strength following 4 weeks of intervention, compared to baseline values. Whiskers signify SE.

MVC data are displayed in Table 2 and Fig. 2. Total leg (sum of bilateral MVC in both directions, ES 0.40), knee flexor (ES: 0.34), and knee extensor MVC (ES: 0.29) increased more prominently in REAL compared to SHAM, although no clear main effect for real rTMS was found for any MVC outcome measure (treatment: *p* > 0.15, treatment × time: *p* > 0.76, time: *p* > 0.23)). In contrast, LEMS increased significantly for REAL but not for SHAM at time of discharge, and increased more for REAL compared to SHAM (*p* = 0.014) (Table 2). LEMS assessments at discharge were performed 71.2 ± 47.2 days after the remaining outcome measures, with no significant difference between groups.

**Table 2:** Objectively and subjectively assessed muscle strength.

	REAL	(*n* = 10)		SHAM	(*n* = 9)		*p*-Values			
Variable	Baseline	Post	Mean change, % (95% CI)	Baseline	Post	Mean change, % (95% CI)	Treatment	Treatment × Time	Time	Effect size (Cohen’s *d*)
Total leg (Nm)	302.0 ± 114.8	365.5 ± 116.9	29.4 (7.4;51.3)	379.8 ± 157.4	416.7 ± 154.8	12.5 (−7.9;32.9)	0.16	0.77	0.28	0.40
Knee extension (Nm)	224.0 ± 92.3	262.1 ± 82.3	28.1 (3.8;52.4)	287.3 ± 142.5	306.0 ± 134.3	10.9 (−9.7;31.5)	0.17	0.80	0.46	0.34
Knee flexion (Nm)	78.1 ± 47.7	103.4 ± 61.6	39.8 (16.4;63.1)	92.5 ± 50.3	110.7 ± 55.4	22.6 (0.9;44.4)	0.55	0.84	0.24	0.29
	Admission	Discharge	Change	Admission	Discharge	Change	Within-group REAL	Within-group SHAM	Between group	
Lower Extremity Motor Score	28 [17;40]	40.5 [33;48]	12.5	42 [27;46]	39 [28.5;36.5]	−3	<0.01^a^	0.22^a^	<0.02^b^	

Data are presented as means ± SD or medians [IQR 25%;75%] unless specified otherwise.

^a^Wilcoxon’s signed-rank test.

^b^Mann–Whitney *U*-test.

### Gait function

**Fig. 3:**
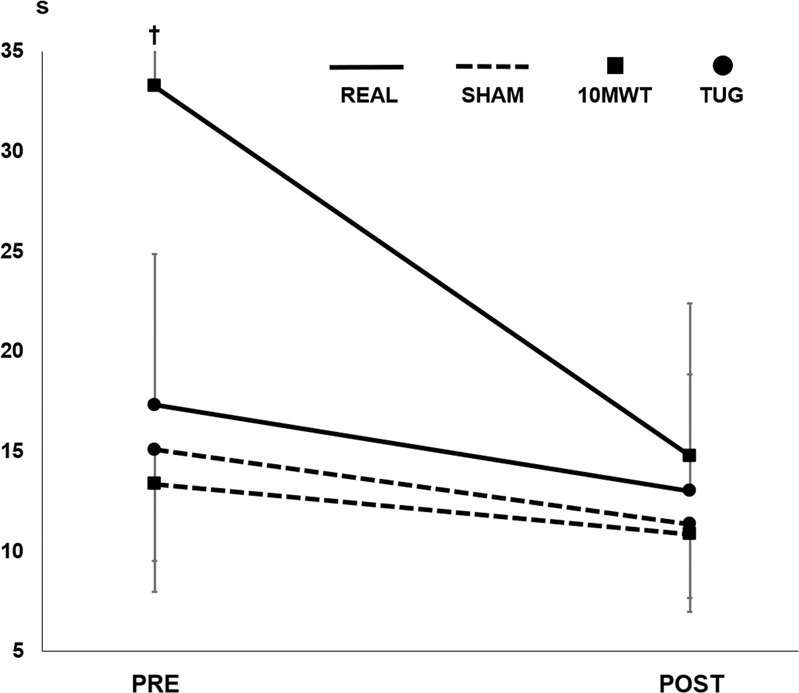
Time-to-complete the 10 m walking test (10MWT) and Timed Up-and-Go (TUG) test before and after 4 weeks of intervention. Whiskers signify SD. †SD = 36.5 s.

Figure 3 depicts group mean data in the 10MWT and TUG test. In 10MWT, both groups demonstrated reduced time-to-complete at POST (REAL 18.5 ± 30.5 s, *p* < 0.01; SHAM 2.5 ± 2.1 s, *p* < 0.02), with no difference in progression between groups (*p* = 0.16). Similar developments were seen for TUG, where both groups reduced time-to-complete (REAL 4.3 ± 3.0 s; SHAM 3.7 ± 3.8 s), but no main effects were seen (treatment: *p* = 0.39, treatment × time: *p* = 0.90, time: *p* = 0.09). Likewise, both groups were able to cover a longer distance in the 6MWT at POST (REAL 77.7 ± 65.5 m; SHAM 75.6 ± 56.9 m) with no clear main effects (treatment: *p* = 0.76, treatment × time: *p* = 0.11, time: *p* = 0.76).

### Adverse effects

One participant in REAL (21-year-old male with incomplete T11 injury), who was otherwise healthy and with no personal or familial history of epilepsy, dropped out due to a seizure during stimulation. In addition, only harmless side effects such as drowsiness, twitching facial muscles, and tingling/poking sensations in the scalp were occasionally reported. Surprisingly, two individuals from SHAM reported mild and transitory headaches following their first treatment session.

## Discussion

This study is the first to investigate the effects of rTMS as adjunct therapy to RT in individuals with SCI. Our results indicate that rTMS is feasible to apply as adjunct therapy during primary rehabilitation, and that it may improve clinically assessed lower limb muscle function. These findings are important, because muscle weakness is a considerable problem for persons with SCI and current therapeutic strategies show variable efficiency.

However, a clear, clinically important difference in short-term recovery of maximal leg muscle strength was not established in the present study. Compared to sham stimulation, real rTMS treatment was associated with greater increase in total leg MVC to an extent beyond that of our predetermined MCID of 15% (63.5 vs. 37.3 Nm increases, Cohen’s *d* = 40% treatment ES), but the 95%CI for the difference spanned as low as −39.7 Nm. The failure to detect any significant differences may partly be explained by the fact that this study was underpowered; due to a high quota of volunteers that were ineligible to participate and recruitment restrictions due to the COVID-19 pandemic, only 19 participants completed the study out of 30 needed for sufficient statistical power. Studies that employ larger sample sizes should be conducted to elucidate the effect of rTMS on maximal muscle strength. Previous findings from individuals with ALS [27] suggests that as little as 2 weeks of high-frequency rTMS can improve total body isometric MVC and lower limb isokinetic mean power significantly more than sham stimulation. Thus, it is possible that for individuals with SCI, the beneficial effects of rTMS on the injured corticospinal tract require a time frame longer than 4 weeks to manifest as clinically relevant improvements.

Indeed, the intervention period employed in the present study may have been too brief for evoking clear RT-induced gains in muscle strength. It is common to employ RT interventions of ≥8–12 weeks when evaluating the adaptive effects of RT in a variety of populations, including neurological patients. For example, Bye et al. [28] investigated the effects of 12 weeks unilateral RT in 30 individuals with SCI. Following training, a more pronounced increase in isometric MVC was observed in the trained limb compared to the untrained control limb. However, it was unclear if this difference was clinically relevant or not. In a case series, Gregory et al. [29] found that 12 weeks of supervised RT performed by three individuals with SCI resulted in a ∼30% increase in maximal isometric knee extensor torque (iMVC_KE_). Studying the effects of a progressive RT regimen of similar length to the one employed in the present study (4 weeks), Jayaraman et al. [30] found no gains in iMVC_KE_ nor in total leg MVC in five males (50 ± 12 years) with chronic (>1 year) SCI (AIS: C–D) post training [29]. In contrast, participants in the present study demonstrated marked increases in iMVC_KE_ (∼28%) and total leg MVC (∼29%) in response to 4 weeks of progressive RT combined with active rTMS. Thus, it is possible that the effects of the present intervention would have been more pronounced if it had been extended beyond 4 weeks.

Further, it is possible that the stimulation intensity used in the present study was too low to elicit marked short-term increases in lower extremity MVC. The present stimulation protocol was designed to induce potent neurophysiological effects in the study population, while still adhering to the international safety recommendations for rTMS [31, 32]. However, due to the complicated nature of SCI, it is possible that higher stimulation intensities are needed to augment the therapeutic efficiency. Nonetheless, all rTMS trials should carefully balance the overall risk/benefit ratio to minimize discomfort for the subjects and to prevent adverse events [32].

In addition, the present intervention protocol lacks control of key training variables. It is known that relative and absolute training intensity (load magnitude), number of repetitions performed, rest periods, and contraction velocity are highly influential on the individual neuromuscular response evoked by RT [33, 34]. For example, in the 4 week SCI training study mentioned above [30], a maximal-intensity RT regimen was also performed. Here, participants performed maximal voluntary effort contractions for each exercise. Following this training condition, subjects showed significant increase in peak lower limb isometric torque levels compared with the conventional training condition. In the present study, the main objective was to investigate the effects of rTMS as adjunct therapy to RT performed as part of usual care. Therefore, RT was carried out in a class environment as part of the usual care protocol, without any interference from study staff. Consequently, even though training followed international guidelines for RT in SCI [19] and total time spent training was similar between groups, actual training volume (load × repetitions) and intensity could have differed between groups. Due to the importance of these variables, future studies should investigate the effects of facilitatory rTMS on the specific progression in these variables during a continued RT regimen in individuals with SCI.

The present study participants achieved similar improvements in gait function regardless of treatment allocation. Notably, rTMS was not delivered in combination with gait training. Thus, it is possible that the effects on gait function had been more pronounced if rTMS had been delivered time-locked with gait training. In support of this notion, Kumru and colleagues [15, 16] reported results from two sham-controlled rTMS studies on individuals with SCI where recovery of gait function was the primary outcome parameter and rTMS was combined with supervised gait training. In their first study [15], significant improvements were found in the TUG for both groups, whereas improvements in 10MWT were observed only with real and not sham stimulation. In their subsequent study [16], the two groups displayed similar developments in gait velocity, cadence, and step length, accompanied by a trend for more participants being able to complete the 10MWT at follow-up with rTMS than sham stimulation. Collectively, the promising but somewhat inconsistent available data indicate that further research should be conducted to make more firm conclusions about the efficiency of adjunct rTMS therapy in improving gait function following SCI.

In conclusion, the clinical relevance of the accentuated improvements in maximal volitional muscle strength induced by rTMS therapy during short-term RT is unclear. However, the results indicate that 4 weeks of adjuvant rTMS therapy during primary rehabilitation from SCI can enhance long-term recovery in clinically assessed lower limb muscle function. In addition, therapeutic rTMS, when delivered in conjunction with short-term RT, had no effect on the recovery in gait function, indicating that a task-specific effect exists for the type of training performed in combination with stimulation. Future studies investigating the role of rTMS in the recovery of maximal muscle strength in SCI should employ longer intervention periods and larger study populations, and should also investigate the potential effects on training capacity and intensity.

## Data Availability

The datasets generated during the current study are available in deidentified form from the corresponding author upon reasonable request and following approval from the Danish Data Protection Agency.
